# Lipoprotein (a)-Related Inflammatory Imbalance: A Novel Horizon for the Development of Atherosclerosis

**DOI:** 10.1007/s11883-024-01215-5

**Published:** 2024-06-15

**Authors:** Ting Qin, Tian-Yi Ma, Kang Huang, Shi-Juan Lu, Jiang-Hua Zhong, Jian-Jun Li

**Affiliations:** 1 Department of Cardiology, Haikou Affiliated Hospital of Central South University Xiangya School of Medicine, Hainan, 570208 China; 2 Cadiometabolic Center, State Key Laboratory of Cardiovascular Diseases, Fu Wai Hospital, National Center for Cardiovascular Diseases, Chinese Academy of Medical Sciences and Peking Union Medical College, Beijing, 100037 China

**Keywords:** Atherosclerosis, lipoprotein(a), Inflammation

## Abstract

**Purpose of Review:**

The primary objective of this review is to explore the pathophysiological roles and clinical implications of lipoprotein(a) [Lp(a)] in the context of atherosclerotic cardiovascular disease (ASCVD). We seek to understand how Lp(a) contributes to inflammation and arteriosclerosis, aiming to provide new insights into the mechanisms of ASCVD progression.

**Recent Findings:**

Recent research highlights Lp(a) as an independent risk factor for ASCVD. Studies show that Lp(a) not only promotes the inflammatory processes but also interacts with various cellular components, leading to endothelial dysfunction and smooth muscle cell proliferation. The dual role of Lp(a) in both instigating and, under certain conditions, mitigating inflammation is particularly noteworthy.

**Summary:**

This review finds that Lp(a) plays a complex role in the development of ASCVD through its involvement in inflammatory pathways. The interplay between Lp(a) levels and inflammatory responses highlights its potential as a target for therapeutic intervention. These insights could pave the way for novel approaches in managing and preventing ASCVD, urging further investigation into Lp(a) as a therapeutic target.

## Introduction

Atherosclerosis (AS) stands as a leading cause of increasing morbidity and mortality among adults worldwide. Despite improvements in traditional treatments, they continue to be associated with a significant number of adverse events. In the context of comprehensive control of other cardiovascular disease (CVD) risk factors, the concept of "residual inflammatory risk" primarily refers to the risk of CVD caused by vascular and/or systemic inflammation [1].The JUPITER study highlights that even in the presence of optimal low-density lipoprotein (LDL) cholesterol levels, lipoprotein(a) [Lp(a)] continues to pose a significant residual risk for cardiovascular events [2].The relationship between Lp(a) and CVD has been extensively examined in major studies over the past decade, which are consolidated in Table 1.

**Table 1:** Major studies on the association of Lp(a) with cardiovascular disease between 2003 and 2023

Study name	Study type	Number of participants	Key findings
Copenhagen City Heart Study (2008) [3]	Observational Study	9,330 men and women	Increased Lp(a) concentrations were significantly and independently associated with an elevated risk of coronary heart disease
INTERHEART Study (2009) [4]	International Case–Control Study	Nearly 30,000 participants	Higher Lp(a) levels were linked to an increased risk of non-fatal myocardial infarction and coronary artery death
Robert Clarke (2009) [5]	Genetic Research	3,100 cases of CHD	Identified two specific LPA SNPs (rs10455872 and rs3798220) strongly associated with coronary heart disease risk
Kamstrup et al. (2012) [6]	Mendelian Randomized Study	41,231 cases	Genetically determined high Lp(a) levels doubled the risk of atherosclerotic stenosis of coronary, carotid, and femoral arteries
PROCARDIS study (2014) [7]	Case–Control Study	Over 4 million cases	A 39% reduction in Lp(a) concentration was associated with a 21% reduced risk of coronary heart disease, suggesting therapeutic potential
Michelle L O'Donoghue et al. (2014) [8]	Observational Study	18,978 subjects	Lp(a) was significantly associated with the risk of cardiovascular events in patients diagnosed with coronary artery disease
Aniruddh P Patel et al. (2021) [9]	Cohort Study	460,506 subjects	Each 50-nmol/L increase in Lp(a) concentration was associated with a 1.11-fold increased risk of ASCVD events or recurrent events

**Fig. 1:**
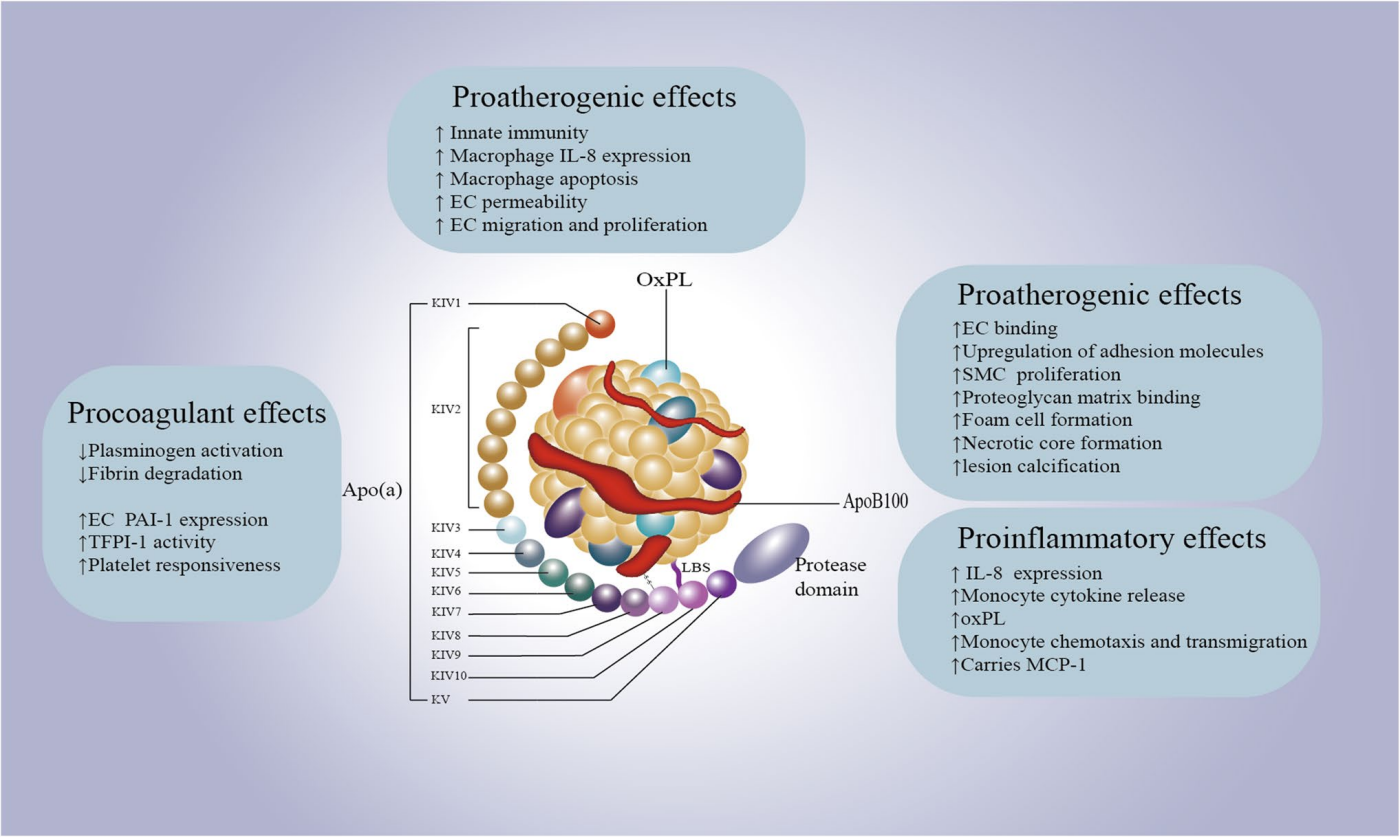
The structure and potential impact of Lp(a) on atherosclerotic processes. Lipoprotein(a) or Lp(a) combines an LDL-like particle with [Apo(a), linked to ApoB100. Its unique structure contributes to cardiovascular disease risks due to procoagulant, proinflammatory, and proatherogenic effects. Elevated Lp(a) levels are associated with increased risk of atherosclerosis and coronary artery disease

Lp(a) resembles LDL in structure and composition, comprising cholesterol esters, phospholipids, and apolipoprotein B100 (ApoB100), alongside the distinctive apolipoprotein(a) [Apo(a)]. Apo(a) uniquely binds to ApoB100 in a 1:1 ratio via disulfide bonds [10–12••, 13]. Unlike ApoB, Apo(a) is hydrophilic and interacts with endothelial cell molecules through its lysine binding site (LBS). In the specific context of human KIV-10, robust LBSs are crucial due to the attachment of oxidized phospholipids (oxPLs), which are key to Lp(a)'s proinflammatory role. Intriguingly, without a strong LBS, Lp(a) in other species fails to attach to oxPLs, a feature unique to human Lp(a). The critical cysteine that forms disulfide bonds with Apo B-100 is located in KIV-9 [14]. The structure and potential impact of Lp(a) on atherosclerotic processes are summarized in Fig. 1.

The genetic makeup determines plasma levels of Lp(a), which are inversely related to the number of KIV2 repeats and remain consistent over a person's life. According to the 2018 AHA/ACC Blood Lipid guidelines and the 2019 ESC/EAS recommendations, it is prudent to measure Lp(a) levels at least once during a lifetime, especially for individuals at elevated risk of ASCVD [15••]. Global clinical practice guidelines show variation in the threshold values for Lp(a), affected by multiple factors. Key among these is genetic diversity, which markedly influences Lp(a) levels as studies reveal significant variations among different ethnicities. For example, African descent populations typically have higher Lp(a) levels compared to those of Asian descent, who usually have lower levels [11]. Furthermore, differences in study designs such as sample population selection, measurement methods, and statistical modeling significantly influence the assessment of risks and the establishment of Lp(a) threshold levels [16]. Variability in disease definitions and risk assessment methods across guidelines contributes to the range of Lp(a) threshold values. Additionally, the diversity in prevention strategies and treatment objectives may necessitate tailored adjustments to Lp(a) thresholds based on the guidelines' specific aims. For instance, guidelines focused on lowering the general risk of cardiovascular diseases often adopt lower Lp(a) thresholds to cover a wider range of patients. Table 2 summarizes the current Lp(a) thresholds established by the guideline committees.

**Table 2:** Lp(a) threshold determined by the Guide Committee

Guideline committee	Year	Lp(a) Threshold
American College of Cardiology/American Heart Association Cholesterol Guidelines (ACC/AHA) [17]	2018	≥ 125 nmol/L (≥ 50 mg/dL)
National Lipid Association Scientific Statement [18]	2019	≥ 100 nmol/L (≥ 50 mg/dL)
European Society of Cardiology/European Atherosclerosis Society Guidelines for the Management of Dyslipidaemias [19]	2019	> 430 nmol/L (> 180 mg/dL)
HEART UK Consensus Statement [20]	2019	> 90 nmol/L
Endocrine Society Lipid Management Guidelines [21]	2020	≥ 125 nmol/L (≥ 50 mg/dL)
Canadian Guidelines for the Management of Dyslipidemia [22]	2021	≥ 100 nmol/L (≥ 50 mg/dL)
Chinese guidelines for lipid management (2023) [23]	2023	≥ 300 mg/L

While the exact process by which Lp(a) leads to atherosclerosis is not fully understood, considerable evidence indicates that Lp(a) can penetrate the arterial wall, enhance cholesterol deposition in the intima, and stimulate endothelial cells, thereby triggering inflammation in the vascular wall [24]. Moreover, the relationship between inflammation and plasma Lp(a) levels is bidirectional, indicating that Lp(a) can display proinflammatory effects in some conditions, whereas in other situations, it might exhibit anti-inflammatory characteristics [25]. Current studies confirm that inflammation impacts Lp(a) levels, with particular inflammatory factors having unique effects on Lp(a). This bidirectional interaction highlights Lp(a)'s critical role in both the onset and advancement of atherosclerosis. This review seeks to explore in depth the ways Lp(a) alters the inflammatory balance and engages with inflammation in the development and progression of AS, while also reviewing relevant clinical data.

### Lp(a)-Related Inflammatory Effect on AS

**Fig. 2:**
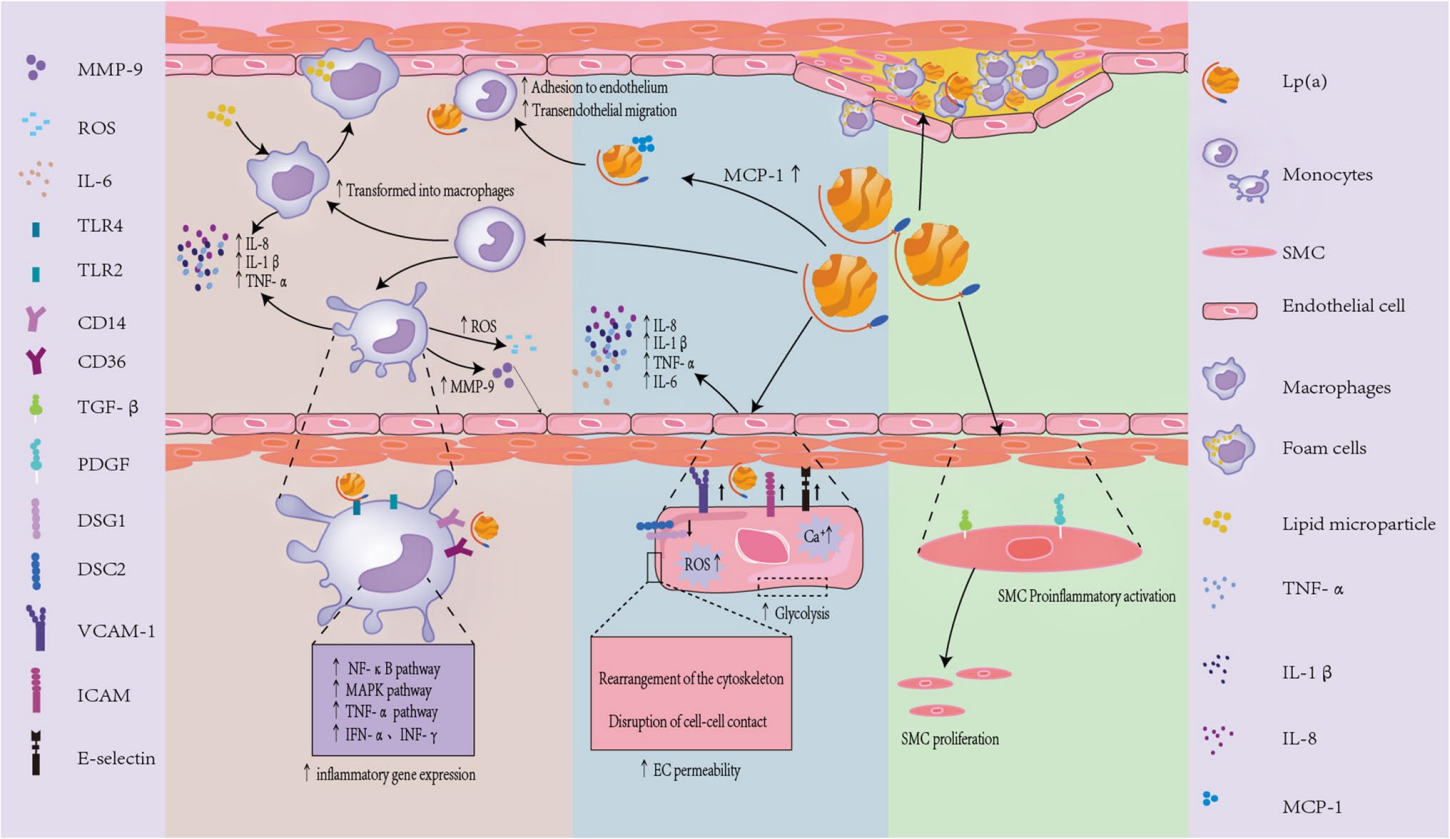
Inflammatory effect process induced by Lp(a). The pro-inflammatory effects of Lp(a) can be broadly categorized into three main mechanisms: activation of monocytes, endothelial dysfunction, and SMC proliferation and phenotypic transformation. (1) Effects on Monocytes: Lp(a) binds to oxPLs, exhibiting strong pro-inflammatory actions. It induces the secretion and attachment of MCP-1, promoting monocyte migration. Additionally, Lp(a) stimulates the secretion of CCL-1 and enhances the expression of β2 integrin-Mac-1, significantly increasing monocyte adhesion to endothelial cells and transendothelial migration, thereby exacerbating arterial wall inflammation. Lp(a) also upregulates inflammatory gene expression in monocytes, promoting their differentiation into macrophages and enhancing cholesterol uptake and metabolism, thus accelerating the development and progression of atherosclerosis. Furthermore, Lp(a) triggers the release of reactive oxygen species and MMP-9, contributing to plaque instability. It also activates TLR2 and TLR4, along with CD14 and CD36, involving inflammatory pathways such as NF-κB and promoting the secretion of cytokines like IL-8, IL-1β, and TNF-α. (2) Effects on Endothelial Cells: Lp(a) activates endothelial cells, significantly increasing the secretion of inflammatory molecules like IL-8, IL-1β, TNF-α, and IL-6, as well as adhesion molecules such as VCAM-1, ICAM, and e-selectins. It causes rearrangement of the endothelial cell cytoskeleton and disrupts adhesion junctions. Additionally, Lp(a) stimulates endothelial cells to produce reactive oxygen species, downregulates the expression of DSG1 and DSC2, altering endothelial cell permeability and causing endothelial dysfunction, which may lead to endothelial cell autophagy. Moreover, Lp(a) enhances glycolysis in endothelial cells, intensifying inflammation within the arterial wall.(3) Effects on Smooth Muscle Cells: Lp(a) inhibits the activation of TGF-β and increases the expression of PDGF, stimulating SMC proliferation and inducing pro-inflammatory activation of SMCs. These mechanisms collectively contribute to the pro-inflammatory environment associated with Lp(a), playing a significant role in the development of atherosclerosis and cardiovascular diseases

Lp(a) is crucial in atherosclerosis, influencing processes like foam cell formation, smooth muscle cell proliferation, and plaque inflammation and instability. Studies show that following endothelial injury, Lp(a) gathers and attaches to different matrix components, initiating the chemotactic activation of monocytes and macrophages, causing endothelial dysfunction, smooth muscle cell (SMC) proliferation, and intensifying local inflammation [26]. An overview of the inflammatory processes induced by Lp(a) is illustrated in Fig. 2.

### Monocyte Activation

Lp(a) facilitates both the chemotaxis and trans-endothelial migration of monocytes. The activation of these monocytes is closely associated with the inflammatory response, which exacerbates the progression of atherosclerosis.

The majority of circulating oxidized phospholipids (OxPLs) are bound to Lp(a). These OxPLs contribute potent pro-inflammatory properties to Lp(a) and are crucial for its functional role. Lp(a) induces the secretion and attachment of monocyte chemoattractant protein 1 (MCP-1), a chemokine crucial for initiating and driving vascular inflammation [27]. Furthermore, oxPLs are identified as the primary components that bind to MCP-1 [27].Within the circulatory system, oxPLs exhibit a strong affinity for binding to Lp(a) [28] and are recognized as danger-associated molecular patterns (DAMPs) by pattern recognition receptors (PRRs) on innate immune cells, leading to a significant pro-inflammatory response. Notably, this response is diminished when oxPLs are neutralized with a specific antibody [29]. These findings suggest that the pro-inflammatory effects of Lp(a) on monocytes are partially mediated by oxPLs.The affinity of oxPLs for Lp(a) is influenced by the structural and functional aspects of both molecules, playing a key role in cardiovascular interactions. Lp(a) is composed of a LDL particle and Apo(a), characterized by a lipid-rich core that facilitates the hydrophobic interaction with oxPLs [30]. Apo(a) features kringle domains akin to plasminogen, which bind electrostatically to the negatively charged oxPLs, enhancing their interaction [31, 32]. OxPLs on Lp(a) are identified by macrophages' scavenger receptors, potentially leading to foam cell formation and atherosclerotic plaque development [30, 33]. While the specific number of oxPLs on each Lp(a) particle can differ, it is notably higher than on LDL particles, underscoring Lp(a)'s role in promoting atherosclerosis [31, 33, 34]. Although the quantification of oxPLs on Lp(a) is ongoing, the evidence suggests a significant presence of multiple oxPLs on Lp(a), which may vary based on the extent of lipid oxidation and specific health conditions [31].

Lp(a) facilitates the migration of monocytes toward the endothelium. Stimulation of human vascular endothelial cells with the Apo(a) component, which includes KIV5 to 10, KV, and the protease domain, leads to an increase in mRNA levels of C chemokine ligand 1 (CCL1). Known for its strong chemotactic properties, CCL1 acts as a powerful monocyte attractant [35]. Studies show that Apo(a), a unique element of Lp(a), enhances the expression of β2 integrin-Mac-1. This increase leads to the activation of nuclear factor kappa B (NF-κB), which strongly encourages the adhesion of monocytes to endothelial cells [36].Vander Valk and team used radiological methods to detect increased arterial inflammation in people with high Lp(a) levels. They found that high levels of Lp(a) facilitate the recruitment of monocytes from the bloodstream to the arterial wall, aiding in their migration [29]. These studies provide definitive proof of Lp(a)'s role in worsening inflammation in the arterial walls. Additionally, Lp(a) and Apo(a) stimulation causes human umbilical vein endothelial cells (HUVECs) and coronary artery endothelial cells (HCAECs) to display enhanced monocyte migration through 5 μM pores in the Boyden chamber [37]. Recent clinical studies on monocyte activation phenotypes have confirmed that Lp(a) facilitates the trans-endothelial migration of monocytes. In the early stages of atherosclerotic plaque development, Lp(a) exacerbates inflammation in the arterial wall by increasing monocyte trans-endothelial migration [38].

Lp(a) significantly contributes to inflammation by enhancing the inflammatory gene expression in circulating monocytes. This upregulation involves multiple mechanisms: first, Lp(a) binds to CD36 receptors on monocytes, triggering MAPK and NF-κB pathways that control inflammatory gene expression. Second, Lp(a) induces oxidative stress in monocytes, causing DNA damage and mutations that further increase inflammatory gene expression [38]. Detailed research has shown that in individuals with cardiovascular disease, high levels of Lp(a) enhance the activation of the TNF-α signaling pathway and interferon response genes in monocytes. To verify Lp(a)'s effects, antisense oligonucleotides were used to reduce its levels in patients, which led to a corresponding decrease in inflammatory gene expression in monocytes. These findings strongly support Lp(a)'s pro-inflammatory role in the innate immune system during cardiovascular disease [39]. After increasing inflammatory gene expression, Lp(a) prompts monocytes to emit inflammatory cytokines like interleukin-6 (IL-6), interleukin-8 (IL-8), and TNF-α. These cytokines then influence other immune cells, including macrophages and T lymphocytes, intensifying the inflammatory response. Simultaneously, Lp(a) encourages monocytes to differentiate into macrophages and enhance cholesterol uptake and metabolism, thus accelerating the development and progression of atherosclerosis [40].

Lp(a) initiates a significant inflammatory response in monocytes by activating Toll-like receptors 2 and 4 (TLR2 and TLR4), along with CD14 and CD36, on monocyte and macrophage surfaces. This leads to the activation of the NF-κB transcription factor, which in turn stimulates IL-8 expression. The activation process also engages ERK (Extracellular Signal-Regulated Kinase) and interacts with JNK (c-Jun N-terminal Kinase) [38].Research has confirmed that blocking the ERK-specific MAPK/ERK (MEK), c-Jun N-terminal kinase, and NF-κB pathways reduces the effects of oxPLs on IL-8 levels, suggesting these pathways mediate this response. Importantly, inhibiting NF-κB significantly decreases the IL-8 expression enhanced by Apo(a) [41].

Lp(a) can trigger the differentiation of inflammatory M1 macrophages, leading to the activation of T helper-1 lymphocytes and natural killer cells. Throughout this process, macrophages release various inflammatory factors, such as interleukin-1β (IL-1β), IL-8, and TNF-α, all of which are stimulated by Lp(a) and contribute to the inflammatory response [40]. Research reveals a link between elevated plasma Lp(a) levels in healthy individuals and increased expression of interferon-α (IFN-α) and interferon-γ (IFN-γ) response genes in monocytes, a connection that diminishes in those with lower Lp(a) levels. Among CVD patients with high Lp(a) levels, there is marked activation of immune pathways, including TNF-α, TLR, IFN-α, and IFN-γ. Additionally, Lp(a) triggers a pro-apoptotic response in ER-stressed macrophages, which intensifies vascular inflammation and may accelerate the transformation of stable atherosclerotic plaques into unstable ones [42].

Research shows that Lp(a) significantly influences monocyte priming in the hematopoietic system of mice. When mouse bone marrow cells were exposed to Lp(a) over a period of 7 days, there was a notable rise in the population of proinflammatory monocytes and macrophages [43]. Monocyte activation is vital in atherosclerotic plaque development. Studies have demonstrated that Lp(a) boosts both the inflammatory and proteolytic capabilities of monocytes, resulting in the release of reactive oxygen species and matrix metalloproteinase-9 (MMP-9). These reactive oxygen species play a role in oxidizing low-density lipoprotein cholesterol into foam cells, and MMP-9 aids in breaking down the extracellular matrix, leading to the rupture of atherosclerotic plaques [44]. Furthermore, monocytes from individuals with elevated Lp(a) levels demonstrate a sustained increased inflammatory response, persisting for at least 7 days, known as the "priming state" [45]. These monocytes are more active in cytokine secretion than those from environments with lower Lp(a). The persistence of this heightened activity remains to be determined, highlighting an area for further study.

After thorough analysis, these studies confirm that Lp(a) is a crucial inflammatory agent. It uniquely initiates intracellular signaling, activates monocytes, and significantly increases their migration to endothelial cells. Together, these actions lead to inflammation of the arterial wall, exacerbating CVD.

### Endothelial Dysfunction

Lp(a) utilizes various mechanisms to induce endothelial dysfunction. These mechanisms work synergistically, collectively enhancing the formation and progression of atherosclerotic plaques.

Lp(a) enhances the expression of pro-inflammatory adhesion molecules in endothelial cells. Initial in vitro experiments demonstrated that Lp(a) stimulates HUVECs to produce vascular cell adhesion molecule-1 (VCAM-1) and E-selectin in a dose-responsive way [45]. Additionally, researchers observed that this response was triggered by increased levels of intracellular free calcium, which could be suppressed using the calcium chelator BAPTA/AM and modulated by competitive interaction with recombinant Apo(a) (r-Apo(a)) [46].Co-incubation of Lp(a) with human aortic endothelial cells (HAECs) significantly increased the secretion of inflammatory cytokines IL-6 and IL-8, compared to endothelial cells without Lp(a). This significant change was confirmed using precise qPCR and Western blotting methods. Additionally, there was an evident increase in the expression of monocyte chemoattractant protein-1 (MCP-1) and adhesion markers like ICAM-1, E-selectin, and VCAM-1 [38]. These results clearly demonstrate that Lp(a) induces a pro-inflammatory response in HAECs, consistent with earlier studies. Additionally, research conducted by Chinese scientists has found a strong link between Lp(a) levels and increased VCAM-1 protein expression [47]. Lp(a) promotes ICAM-1 expression in HUVECs and this increase is linked with decreased activity of transforming growth factor β (TGF-β) [45]. TGF-β, a multifunctional immunomodulatory cytokine, is crucial for maintaining peripheral immune tolerance [48]. The findings imply that Lp(a) could play an indirect role in immune regulation by influencing TGF-β activity.

Lp(a) could double the adhesion rate of monocytes and lead to a fivefold increase in their trans-endothelial migration towards endothelial cells treated with Lp(a) [38]. Furthermore, researchers observed an interaction between the Apo(a) KIV domain and the β2 integrin Mac-1, which activated NF-kB. This activation resulted in increased monocyte adhesion to the endothelium and their infiltration into the arterial wall [36]. Lp(a) potentially accelerates atherosclerosis progression by triggering the expression of adhesion molecules on endothelial cells, which enhances leukocyte attachment to the vascular walls. The recruitment of these white blood cells is a key initial step in the development of atherosclerosis. Therefore, Lp(a)'s ability to activate endothelial cells marks a pivotal point in the early stages of atherosclerotic disease, enriching our comprehension of Lp(a)'s impact on atherosclerosis [47].

Lp(a) induces pro-inflammatory effects by rearranging the cytoskeleton and disrupting adhesion junctions, which impairs endothelial integrity. The Apo(a) component of Lp(a) influences the Rho and Rho kinase signaling pathways, initiating changes that increase endothelial cell permeability. This multifaceted process involves forming f-actin stress fibers, creating junction gaps, and breaking down cell–cell contacts via ve-cadherin degradation [49, 50]. Simultaneously, Apo(a) binds to the lysine binding site in KIV(10') of Rho kinase, leading to the inhibition of myosin light chain (MLC) phosphatase. This inhibition causes the phosphorylation of myosin light chain, further impacting cell structure [51]. These responses involve not only the formation of actin stress fibers and cytoskeleton rearrangement but also affect endothelial cell permeability, ultimately compromising their barrier function [25]. Beyond the Apo(a)/Rho/VE-cadherin pathway, reactive oxygen species (ROS) play a crucial role in affecting endothelial cell permeability [52]. In certain experiments, copper sulfate (CuSO4) was used to oxidize Lp(a), leading to ROS generation in HUVECs. These ROS decrease the transcription levels of adhesion molecules like desmocolin-1 (DSG1) and desmocolin-2 (DSC2), impacting endothelial permeability [53]. Additionally, copper sulfate-oxidized Lp(a) triggers the conversion of LC3-I to LC3-II and increases beclin-1 expression in HUVECs via the PAPR-1-LKB1-AMPK-mTOR pathway, which promotes autophagy in these cells [54].

Studies have shown that Lp(a) boosts glycolysis in endothelial cells, driven by fructose-6-phosphate-2-kinase/fructose-2,6-bisphosphatase 3 (PFKFB3), and triggers inflammation. Under Lp(a) stimulation, HAECs exhibited increased glycolytic activity and higher expression of related genes and proteins. This activity also led to increased secretion of metabolic byproducts such as glucose-6-phosphate, pyruvate, succinic acid, fumaric acid, and lactic acid. Further research demonstrated that inhibiting PFKFB3 reduced inflammation and cell migration in these endothelial cells. This suggests that PFKFB3 is crucial in Lp(a)-driven vascular inflammation and points to potential therapeutic strategies targeting endothelial glycolysis to reduce arterial wall inflammation. Importantly, these findings indicate that the inflammatory effects of Lp(a) on endothelial cells are partially reversible [38].

### Proliferation and Pro-Inflammatory Activation of SMC

Vascular smooth muscle cells (VSMCs) are highly adaptable, with phenotypes that change dynamically in response to different environmental factors. In the course of atherosclerosis, VSMCs transition into a distinct "synthetic" and "pro-inflammatory" phenotype. This transformation leads them to release chemokines and cytokines, crucial for regulating monocyte/macrophage infiltration, thus significantly enhancing vascular inflammation [55].Research indicates that Lp(a) facilitates the proliferation of smooth muscle cells (SMCs). It has been found to boost SMC growth in the vascular wall by both inhibiting the activation of TGF-β [56] and elevating the levels of platelet-derived growth factor (PDGF) [57] from endothelial cells. Moreover, LDL particles contained in Lp(a) also directly encourage SMC proliferation [58]. In research by Komai et al., the growth-promoting effects of Lp(a) and its oxidized form on human VSMCs were evaluated. The study showed that Lp(a) significantly stimulates VSMC proliferation in a dose-responsive manner. Notably, oxidized Lp(a) was found to have a more potent effect on VSMC growth than its natural counterpart. The extracellular signal-regulated kinase (ERK) pathway was crucial in facilitating these effects [59].

Lp(a) is implicated in the pro-inflammatory activation of smooth muscle cells (SMCs). MIAT, a long non-coding RNA, plays a crucial role in the progression of advanced arteriosclerosis, with its expression elevated by higher levels of Lp(a). This increase leads to SMC proliferation via the ERK-ELK1-EGR1 pathway, activates the NF-κB pathway enhancing macrophage inflammation, and boosts KLF4 activity, pushing SMCs towards a macrophage-like inflammatory phenotype, thus exacerbating vascular inflammation [60]. Additionally, Lp(a) raises α 7-nAChR levels in HCASMCs from CAS patients, with Lp(a) and α 7-nAChR jointly activating M6 macrophages and HCASMCs via the p1MAPK/IL-38/RhoA-GTP pathway. Treatment with Topirazumab, an IL-6 receptor-targeting antibody, lessens α 7-nAChR activation and lowers levels of p38MAPK, IL-6, and RhoA-GTP in HCASMCs [61]. These findings elucidate how Lp(a) contributes to vascular dysfunction and CAS development, highlighting the potential of targeting specific pathways to alleviate these effects. Further research is needed to fully understand Lp(a)'s role in SMC phenotypic changes.

### Anti-Inflammatory Effect of Lp(a) and AS

As inflammation and Lp(a) research evolves, a theory of bidirectional effects is gaining acceptance. Research shows Lp(a) can trigger pro-inflammatory responses at both molecular and cellular levels, yet it also might exert anti-inflammatory effects under certain disease states. This perspective provides fresh insights into Lp(a)'s complex role in various diseases.

Up to 90% of all oxPLs in human lipoproteins are transported by Lp(a), highlighting its role as the primary carrier [34, 62]. This function is vital for Lp(a)'s role in the circulatory system, helping to remove oxPLs and potentially reducing inflammation related to oxidative stress [34].

Present studies indicate that oxPLs manifest dual roles, exhibiting both pro-inflammatory and anti-inflammatory activities based on indirect action mechanisms [63]. Specific lipid mediators, created through radical-initiated peroxidation and enzymatic processes, generate oxPLs which inhibit Toll-like receptor triggering by external microbial elements and interfere with the activation of the pro-inflammatory factor NF-kB, thus manifesting anti-inflammatory properties [64]. While complete oxPL molecules possess anti-inflammatory qualities that guard against inflammatory disorders, the truncated versions intensify inflammation and advance inflammatory disease progression [65]. The function of oxPLs as pro-inflammatory or anti-inflammatory agents also hinges on their concentration within a locale; they act as anti-inflammatory agents at lower levels but assume pro-inflammatory roles at higher concentrations [66, 67]. Further, the latest studies reveal that the anti-inflammatory efficacy of oxPLs is predominantly driven by those containing cyclopentenone. These molecules have proven effective in alleviating inflammation in living organisms, bearing significant resemblance both functionally and structurally to natural prostanoids, hence replicating their biological behaviors [68]. Despite common beliefs attributing oxPLs to the enhancement of Lp(a)-related atherosclerosis, their possible anti-inflammatory properties suggest a beneficial role in tempering inflammatory effects linked to Lp(a).

Lp(a) itself may also have certain anti-inflammatory effects. For example, during the process of lipopolysaccharide-induced endotoxemia, lipoproteins can play a neutralizing role, exerting a direct anti-inflammatory effect [69]. In two inflammatory models, namely sodium thioglycolate-induced peritonitis and CaCl2-induced abdominal aortic aneurysm, Apo(a) effectively suppressed neutrophil recruitment by inhibiting cytokine release and reducing neutrophil entry into the vascular wall [70]. However, it is important to note that the same study also found that Lp(a)/Apo(a) inhibited the recruitment of inflammatory cells while significantly increasing the count of white blood cells. Whether this situation might be potentially harmful requires further consideration and investigation [25].

Lp(a) and oxPLs and their impact on inflammation responses are complex and variable. These complex interactions remind us that we need to be more meticulous and comprehensive when developing therapeutic strategies for cardiovascular diseases.

### Lp (a) Levels and Inflammation

While plasma levels of Lp(a) are primarily influenced by genetic factors, certain studies indicate that inflammation could potentially impact the expression and plasma levels of Lp(a). Elevated levels of lipoprotein(a) have been observed in various chronic inflammatory conditions like lupus, as well as in acute inflammatory situations such as post-surgery [71, 72]. This article will delve into the discussion of how inflammatory markers associated with AS and CVD can affect levels of lipoprotein(a).

Inflammation is key in the pathology of ASCVD, with the IL-1β, IL-6, and C-reactive protein (CRP) signaling pathway being central to this role. These inflammatory cytokines are significant markers for cardiovascular disease risk, having a direct and independent causal relationship with the disease [73, 74]. The relationship between Lp(a) and IL-6 has become a focal point of research. Lp(a)'s gene structure includes IL-6 response elements, which create a direct and distinctive connection between Lp(a) and IL-6. This link enhances our comprehension of cardiovascular disease mechanisms and suggests that targeting IL-6 could be valuable in preventing and treating CVD [75]. Müller et al. substantiated the link between IL-6 and LPA gene expression by incubating human hepatocytes with IL-6. Their findings demonstrated that IL-6 upregulates LPA expression via STAT3 binding to the LPA promoter [76]. Tocilizumab, an anti-inflammatory medication, has shown effectiveness in lowering IL-6 and CRP levels in patients experiencing non-ST-segment elevation myocardial infarction. Despite this, Lp(a) levels remained largely unchanged during a six-month follow-up, indicating that more intricate strategies may be needed to reduce Lp(a) levels effectively [77, 78].

Recent studies have shed light on the connection between IL-1β and cardiovascular disease, particularly focusing on how IL-1 genotypes affect cardiovascular risks in patients with elevated Lp(a) levels undergoing angiography. Results revealed a strong link between the IL-1( +) phenotype and elevated Lp(a) levels (over 9.2mg/dL), suggesting these patients are at an increased risk for cardiovascular events. This underscores the potential need for targeted management of Lp(a) levels and IL-1 genotypes to prevent cardiovascular incidents in certain populations [79]. Further studies are essential to fully understand this relationship, especially to determine if the association is independent of other cardiovascular risk factors.

### Therapeutic Frontiers on Lp(a) and Inflammation

While high Lp(a) levels are recognized as a major risk factor for CVD, no targeted treatments currently exist to reduce Lp(a) levels [12••]. Research efforts to decrease lipoprotein(a) have mainly explored its metabolic pathways. Although statins are crucial in lowering LDL-C and preventing ASCVD, they fail to reduce Lp(a) levels; rather, they may increase them. A meta-analysis of 5256 individuals found that Lp(a) levels rose by 8.5% to 19.6% following 12 weeks to 2 years on statin therapy. This increase is not well understood but may involve statins enhancing LPA mRNA and Apo(a) expression [80].

Several new classes of lipid-lowering medications have shown potential in impacting plasma Lp(a) levels. These drugs offer more specific strategies for managing Lp(a) levels and potentially reducing cardiovascular risk.

### PCSK9 Inhibitors

Clinical studies indicate that PCSK9 inhibitors can decrease Lp(a) levels by about 20% to 30% [81, 82]. Specifically, after 48 weeks on Evolocumab, a median Lp(a) reduction of 26.9% was observed. For patients with initial Lp(a) levels above 37 nmol/L, PCSK9 inhibitors can lower the risk of ASCVD by 23% [83]. Furthermore, Inclisiran, the first PCSK9siRNA, reduces Lp(a) levels by roughly 19% to 22% [84]. This data highlights that individuals with elevated initial Lp(a) levels may particularly benefit from reductions in Lp(a).

PCSK9 inhibitors function by blocking the translation of PCSK9 mRNA, thereby decreasing PCSK9 protein production. This action boosts the LDL receptor's (LDLR) ability to clear Lp(a) from the bloodstream [83]. However, while these inhibitors reduce both LDL cholesterol (LDL-C) and Lp(a) levels, specific clinical evidence detailing the impact of Lp(a) reduction alone on ASCVD remains insufficient [81, 82].

Three PCSK9 inhibitor drugs are currently on the market: Evolocumab [85], Alirocumab [86], and Inclisiran [87]. Evolocumab and Alirocumab function as monoclonal antibodies that directly target PCSK9 proteins, whereas Inclisiran sodium acts on PCSK9 by targeting its messenger RNA. While these drugs show promise in reducing Lp(a) levels, more clinical studies are necessary to confirm their effectiveness and safety for this specific use.

### Antisense Drugs

Pelacarsen (AKCEA-APO(a)-LRx), a novel drug targeting high Lp(a) levels, is under development using antisense oligonucleotide technology to inhibit Apo(a) synthesis and reduce Lp(a) concentrations. Clinical trials have already shown Pelacarsen to be safe and well-tolerated, with significant Lp(a) reductions up to 72% observed at various dosages [88]. Additionally, the ongoing Phase III HORIZON trial aims to assess its effectiveness in cardiovascular disease patients further. This large-scale study plans to enroll 7680 patients, randomly assigned to receive Pelacarsen or a placebo. Pelacarsen is expected to reduce Lp(a) levels by up to 80%, aiming to lower average concentrations to 20mg/dl. The outcomes of this pivotal trial, expected in 2024, could make Pelacarsen a new therapeutic option for managing cardiovascular diseases [89].

Mipomersen, an antisense oligonucleotide therapy, targets and reduces apoB100-containing lipoproteins, including Lp(a). A Phase III trial showed that a 26-week regimen of 200mg mipomersen decreased Lp(a) levels by 26.4%. Despite its efficacy, mipomersen is associated with several side effects, including injection-site reactions, hepatic steatosis, and elevated liver enzymes. Additionally, there's no demonstrated reduction in ASCVD events with its use. Consequently, mipomersen is specifically prescribed for familial hypercholesterolemia patients [90].

### Lipoprotein Apheresis

Lipoprotein apheresis (LA) effectively reduces plasma Lp(a) levels and ameliorates blood flow abnormalities, while also decreasing inflammatory factors and the apoE4 subtype. With specific antibody adsorption columns, Lp(a) decreases by approximately 75%, and high-sensitivity C-reactive protein, an inflammatory marker, drops by around 40% [91]. In patients with stable ischemic heart disease, 18 months of lipid apheresis therapy has shown significant stabilization and regression of coronary and carotid artery atherosclerotic lesions [91]. The FDA approves LA treatment when Lp(a) and LDL-C levels surpass certain thresholds in patients who continue to experience coronary atherosclerosis progression despite lipid-lowering drug therapy. Despite its effectiveness, LA is expensive, time-intensive, and is not a standard treatment option for all patients with elevated Lp(a) due to these drawbacks [92].

## Conclusion

Based on the information provided, it's clear that Lp(a) is a structurally complex molecule with unique biological functions. It plays a significant role in the inflammatory process through various mechanisms, contributing to endothelial dysfunction, monocyte and macrophage activation, and smooth muscle cell proliferation, all of which promote the development of AS. It's important to note that there exists a complex bidirectional relationship between Lp(a) and inflammation; Lp(a) can both induce and potentially have anti-inflammatory effects in certain situations. Additionally, inflammation can also influence the levels of Lp(a). Current therapies such as antisense oligonucleotides and siRNA show great promise in significantly reducing Lp(a) levels and are not required to be isoform-specific, given their mechanism of targeting RNA synthesis. Therefore, it is crucial to screen for Lp(a) levels and explore more effective methods to lower them. This research holds promise for offering new strategies for the treatment of atherosclerosis.
